# Study on Thin Lamination of Carbon Fiber Based on Mechanical Broadening

**DOI:** 10.3390/ma17051103

**Published:** 2024-02-28

**Authors:** Yanlei Chen, Yanbo Hui, Dengjie Zhu, Xingle Li, Hongxiao Wang

**Affiliations:** School of Mechanical and Electrical Engineering, Henan University of Technology, Zhengzhou 450001, China

**Keywords:** continuous carbon fiber, mechanical widening, thin lamination, multi-factor, widened defect

## Abstract

Carbon fiber has excellent mechanical properties and plays an important role in modern industry. However, due to the complexity of the carbon fiber widening process, the industrial application of carbon fiber is limited. By designing the carbon fiber widening equipment of automaton, the relationship between the widening width of carbon fiber and the process parameters is studied, and the optimum developing process parameters are obtained, to improve the performance of carbon fiber composites to a certain extent. In this study, the widening process of carbon fiber was studied based on the mechanical broadening method. Firstly, an automatic broadening equipment was designed, and the effects of the initial tension, the number of straight rods, the number of convex rods, and the drawing speed on the widened width during the broadening process were discussed. The widening effect was evaluated by SEM imaging and mechanical testing. At the same time, the factors affecting the broadening width and broadening defects during the broadening process were analyzed, and the optimal broadening process parameters were obtained. The results showed that within a specific range, a higher initial tension, a greater number of convex rods, and an appropriate speed resulted in relatively smaller damage to the broadening of carbon fibers. Through the design of automatic broadening, this experiment explores optimal broadening process parameters, provides a reference for the improvement of the carbon fiber broadening process and further promotes large-scale industrial applications of carbon fiber.

## 1. Introduction

Carbon fiber, as a reinforcing phase in fiber-reinforced composite materials, has a wide range of application scenarios [1,2,3,4]. Despite exceptional mechanical properties exhibited by carbon fiber composites, the matrix penetration effect in the carbon fiber is not ideal during the preparation of composite materials, resulting in easy production of gaps and defects between individual filaments [5,6]. At the same time, the diameter of tow fibers is also prone to fiber buckling and the dislocation of the layering angle during the molding process, resulting in mechanical properties that cannot meet design requirements. Carbon fiber composite structures made of thin layers of carbon fiber prepreg tow have excellent static and dynamic mechanical properties [7,8,9,10]. However, the difficulty and the cost of preparing thin-layer carbon fibers have become a difficult problem restricting the development of high-performance carbon fiber composites [11,12,13]. Therefore, the development of a widening technology for thin lamination of carbon fiber can effectively solve the above problems, improve the performance of the material and promote the large-scale application of thin-layer carbon fiber prepregs.

To ensure the integrity of the mechanical properties of fiber bundles, the automatic stretching technology is used to reduce the thickness of carbon fiber bundles and increase their width, to obtain a thin layer of carbon fiber. The commonly used widening techniques mainly include the mechanical widening method, the ultrasonic widening method, the electrostatic widening method, and the air flow widening method [14]. Studied on the use of ultrasonic widening to expand carbon fibers have been conducted by relying on the effect of ultrasonic vibration to expand carbon fibers. Ma’s group [15] from Jiangnan University invented a widening method that combines ultrasonic and chemical solutions. In the above research, the ultrasonic widening method is inseparable from the chemical solution, and the chemical solution needs to be prepared according to the sizing agent on the surface of carbon fiber, which has relatively high cost and thus a low economic benefit. There are also studies using the method of electrostatic widening to charge carbon fibers, relying on the method of mutual repulsion of the same charges to thin carbon fibers. Ren et al. [16] used the electrostatic widening method to carry out the secondary widening of fiber bundles. The results showed that the high-voltage electrostatic field widening is conducive to the dispersion of fiber bundles and at the same time reduces the abrasion and fracture of fiber bundles during the widening process. However, the electrostatic method needs to remove static electricity from the fiber, and the fiber is prone to problems such as yarn. Some studies have used the method of air flow [17] for widening, relying on the effect of air flow on the carbon fiber, and the fiber bundle becomes curved. The hot air flow melts the paste on the surface of the carbon fiber, weakening the bonding effect between the fiber monofilaments. Due to different air flow rates at different locations, the carbon fiber exerts a transverse force, and the tow becomes thinner. Niu used a two-stage airflow widening device [18] to stretch the carbon fiber tow. Huang’s group [19] used a four-stage air flow stretcher to expand the CF tow step by step, and the fiber bundle width gradually increases with the increase of the stretcher. The air flow widening method can ensure no damage to CF while widening, but it needs to accurately control the size of the air flow and design a suitable air flow stretcher. In the mechanical widening method, a heating roller is used to melt the paste on the surface of the carbon fiber to reduce the bonding effect between the monofilament fibers, and the friction between the fiber and the widening roller or the widening rod is used to expand the CF filament bundle. Scholars such as Ma’s group invented a method to regulate the heating of CF filaments by regulating the contact area of a CF filament bundle on a heating roller and at the same time to expand the CF filament bundle under the action of the yarn guide roller rolling. Karl Meyer (China) Co., LTD [20] uses the conductivity of CF to provide heat by electrifying a CF bundle, thereby removing the sizing agent from the CF surface. Park et al. [21] successfully achieved the expansion of 12 K carbon fiber from 7 mm to 20 mm by employing a self-designed tow spreading device under optimized process conditions. The mechanical widening method has the characteristics of simple device structure and low cost, but if the friction is too large, it is easy to damage the CF tow and reduce the mechanical properties of the prepreg prepared later. Compared with the other four widening methods, the mechanical widening method has the advantages of simple structure, low cost, and easy improvement later, so this study chooses the mechanical widening method.

In this study, the relationship between the broadening width of carbon fiber and the process parameters was studied by using the mechanical broadening method. The impacts of four parameters, namely the initial tension, the quantity of straight rods, the quantity of convex rods, and the winding speed, on the width of broadening were investigated through the establishment of an automated broadening platform. Experimental results were analyzed using an innovative regression model, and the broadening effect was assessed through SEM imaging and mechanical testing. Furthermore, a comprehensive investigation of the factors influencing width and defects in the broadening process was conducted, leading to the determination of optimal parameters for achieving +effective broadening. This study provides valuable insights for enhancing the carbon fiber broadening process, improving the properties of carbon fiber composites and facilitating their widespread industrial application.

## 2. Materials and Methods

### 2.1. Widening Process, Equipment, and Materials

**Figure 1:**
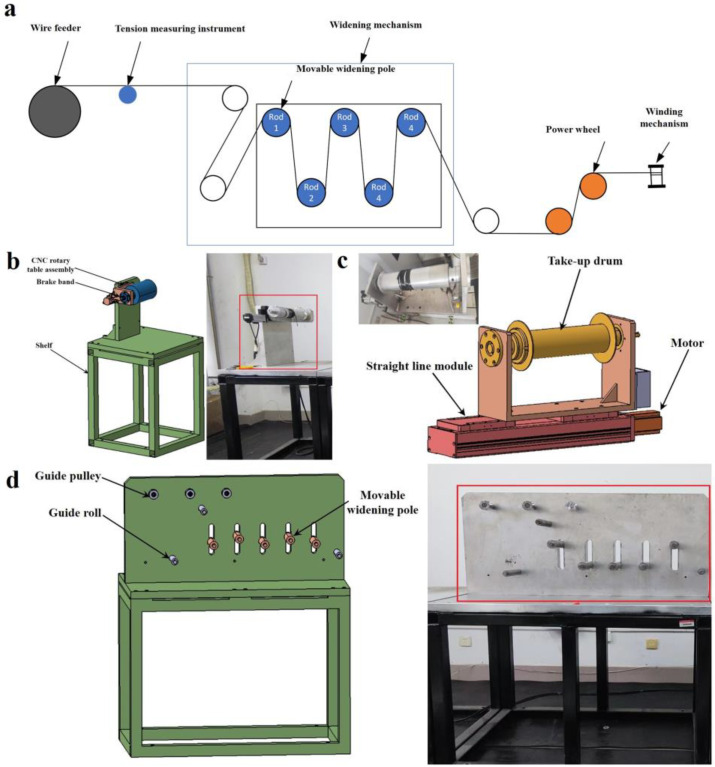
(**a**) CF tow widening process diagram. (**b**) 3D model and picture of the wire feeding mechanism. (**c**) 3D model and picture of the winding mechanism. (**d**) Widening mechanism’s 3D model and picture.

The diagram of the continuous carbon fiber widening process is shown in Figure 1a, and the process parameters in the continuous carbon fiber widening process were accurately regulated by this equipment. In the equipment, a carbon fiber was first sent out by the wire feeding mechanism, as shown in Figure 1b; the initial tension of the carbon fiber was measured by a tension tester. The tension could be adjusted by the wire feeding mechanism. Then, the carbon fiber was broadened through a widening device composed of five movable widening rods that could move up and down, as shown in Figure 1d; finally, the prepreg belt was collected by the winding structure that provided power and rotation during the widening process, as shown in Figure 1c.

In this study, the continuous carbon fiber, branded as TZ700S and consisting of a total of 12 K filaments, was manufactured in Weihai, China. The detailed parameters are shown in Table 1.

### 2.2. CF Tow Pretreatment

The high-temperature cracking method was used to desize the carbon fiber to remove the sizing agent of the epoxy resin system on the surface of the tow, so as to facilitate the expansion of the CF. A muffle oven (SX2-2.5-12, JIE CHENG, Shanghai, China) was used to keep the carbon fiber warm, and the carbon fiber was baked for 30 min, cooled to room temperature and desized.

### 2.3. Damage Analysis of CF Desizing

The tensile test was conducted using an electronic universal testing machine (Instron-5584, INSTRON CORPORATION, Boston, MA, USA) at a speed of 1 mm/min. Typical engineering displacement−load curves were collected to evaluate the damage caused by desizing temperature to CF. An appropriate desizing temperature was selected according to the degree of damage.

In the process of the tensile experiment of the CF tow, many factors affected the accuracy of experimental data to varying degrees, resulting in a certain degree of deviation of tensile data. Therefore, the standard deviation, the performance dispersion coefficient, and the average value were used to reflect the dispersion degree of data. The tensile strength of CF tow was expressed by Formula (1), and the strength damage ratios of CF under different conditions was calculated by Formula (2) [22]:

(1) $$\sigma =\frac{P\rho }{t}$$

where

*σ* is the tensile strength (MPa);
*P* is the failure load (N);
*ρ* is the CF volume density (g/cm^3^);
*t* is the CF line density (g/m).

(2) $$K=\frac{\sigma_{0}-\sigma_{1}}{\sigma_{0}}$$

where

*σ*_0_ is the CF tow tensile strength (MPa);
*σ*_1_ is the tensile strength of specimens under different conditions (MPa);
*K* is the tensile strength damage ratio.

### 2.4. Widening Experimental Scheme Design

The process parameters that affect the broadening effect of CF tow include the initial tension, the number of straight rods, the number of convex rods, the winding speed, etc. To explore the effects of these process parameters on broadening, an orthogonal experiment (*n* = 3) in Table 2 was designed to measure the widening width under each process parameter, which were compared.

The CF monofilament used in this paper was 7 μm in diameter, and the monofilament could be stretched to 84 mm according to the juxtaposed compact arrangement. Therefore, the ratio of the expanded CF bundle width to the theoretical developed width was defined as an effective broadening rate, as shown in Formula (3):

(3) $$\Phi =\frac{w_{a}}{w}$$

where

*w*_a_ is the width after expansion (mm);
*w* is the theoretical broadening width (mm);
*Φ* is the effective broadening rate (%).

### 2.5. Morphological Analysis of CF after Broadening

An inverted microscope (HDMI3860, ALL WAYS, Hangzhou, China) was used to observe the CF tow before and after desizing and after broadening and to observe the desizing and broadening effects under microscopic conditions. To better observe the surface microstructure of the broadened CF fibers, we applied a thin layer of resin to the surface of the samples with different broadened widths to fix the fibers. At the same time, an unbroadened CF filament (6 mm) and the successfully broadened CF filament (12 mm, 15 mm, and 18 mm samples) were sliced and sputtered with a palladium layer. The broadening effect of the surface was observed by scanning electron microscopy (SEM, Sigma 300, Zeiss, Oberkohen, Germany).

### 2.6. Statistical Analysis

All data in this study were expressed as mean ± standard deviation, and one-way ANOVA was used for statistical analysis. For *p* < 0.05, the difference was significant (*), and for *p* < 0.0001, the difference was very significant (**).

## 3. Results and Discussion

### 3.1. Morphological Analysis of Carbon Fiber Desizing and Broadening

**Figure 2:**
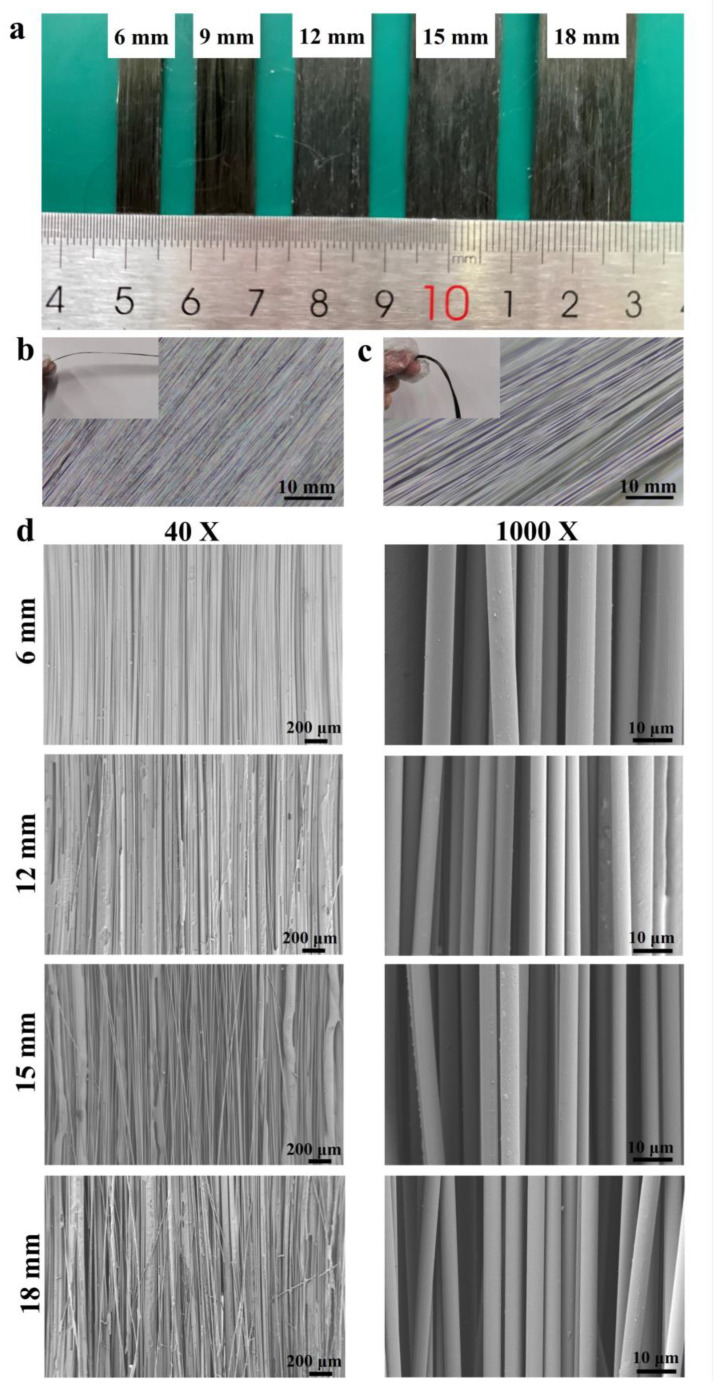
(**a**) Macro-photo of the CF tow widening with different widens. (**b**) Optical microscopic image of the CF before widening and desizing. (**c**) Optical microscopic image of the CF after widening and desizing. (**d**) Surface SEM images of stretched and unstretched CF filament bundles at different magnifications. (The 6 mm sample was the undesized CF tow).

Figure 2a shows a macro-photo of the CF tow under different broadening widths, in which the 6 mm sample was the unbroadened CF tow. Figure 2b,c are the optical microscope images of the CF before and after desizing, respectively. It can be seen from the images that the fiber bundles converged as a whole and the monofilaments were closely arranged in the fiber bundles in terms of surface morphology. The surface of the CF after desizing at high temperature presented a fluffy state, the internal CF filaments appeared relatively independent, and the film layer disappeared, indicating that the material was completely desized. Figure 2d shows the SEM images of CF filament bundles of different spread widths. The fiber bundles of the 15 mm broadened sample had partial entanglement, while the fiber bundles of the 18 mm broadened sample had more serious entanglement and local breakage. The surface of the 12 mm broadened sample was flat, the number of strands with the same magnification was significantly reduced, and the monofilaments were relatively independent, indicating that the broadening effect was good.

### 3.2. Effects of the Widening Process Parameters on the Widened Width

The effects of the broadening factors on the broadened width were studied. To expedite the determination of optimal process parameters influencing the widened width, enhance experimental efficiency and minimize unnecessary trials, the Box–Behnken design was employed for ascertaining the optimum values of response variables [23]. If the number of convex rods in each group of experiments remained unchanged, Box–Behnken experiments were conducted by changing the initial tension, the number of straight rods, and the winding speed. The values of Box–Behnken experimental factors are shown in Table 3. Among them, symbol A represents the initial tension; symbol B_1_ represents the number of straight rods when the number of convex rods is 1; symbol B_2_ represents the number of straight rods when the number of convex rods is 2; symbol B_3_ represents the number of straight rods when the number of convex rods is 1; symbol C represents the winding speed.

**Figure 3:**
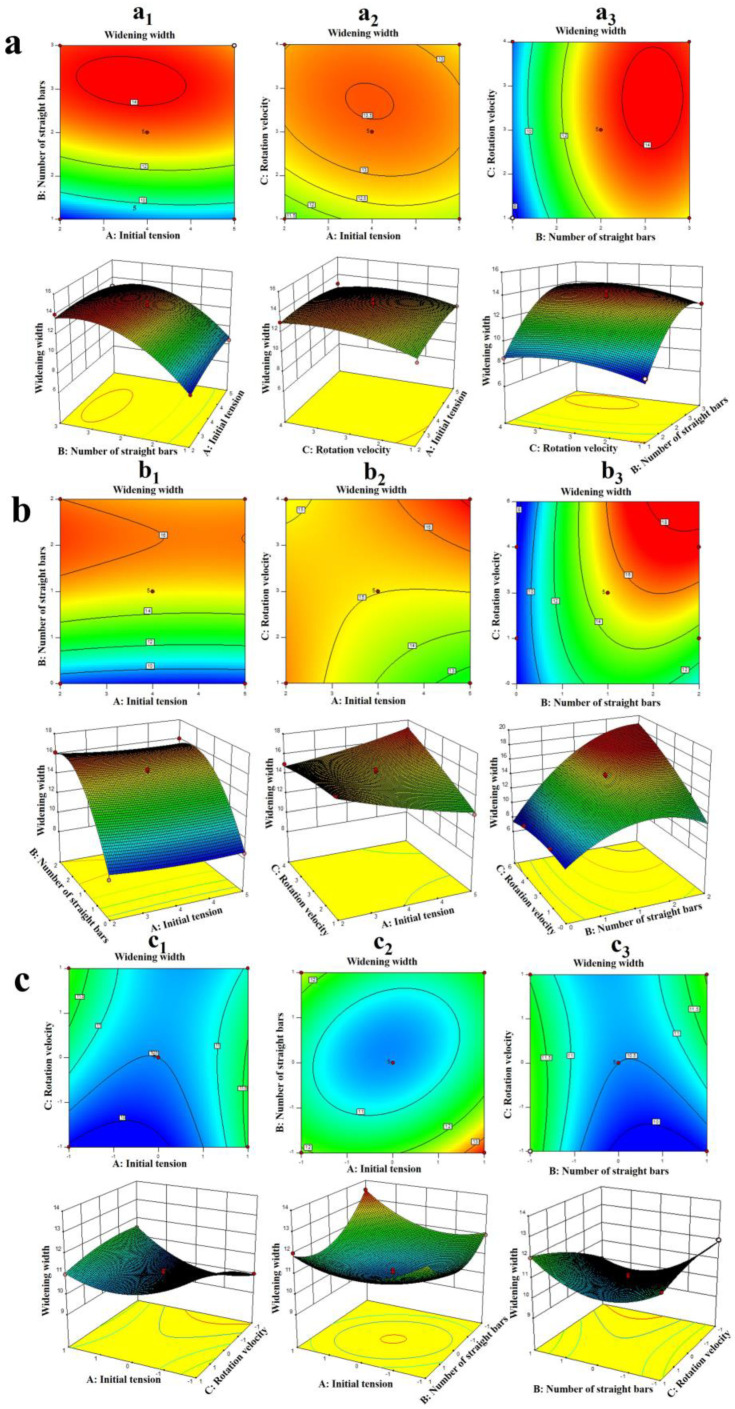
Contour plot and response surface plot of each interaction against the broadened width. (**a**) The number of convex rods is 1; (**b**) The number of convex rods was 2; (**c**) The number of convex rods was 3.3.3. Effects of the Broadening Process Parameters on Broadened Defects.

Regression equations with different numbers of convex rods, Box–Behnken test results, model parameters, and Box–Behnken regression model ANOVA are shown in Table 4, Table 5, Table 6, Table 7, Table 8, Table 9 and Table 10. According to the model, contour maps and response surface maps of two interactive factors related to the initial tension, the winding speed, and the number of straight rods were obtained, and the effect of the interaction between the two factors on the broadened width was observed, as shown in Figure 3.

#### 3.2.1. The Number of Convex Rods was 1

When the number of convex rods was 1 and the winding speed was a constant value, the CF broadened width first increased and then decreased with the increase of the initial tension and the number of straight rods, but the interaction between *A* and *B*_1_ had no significant effect on the broadened width, as shown in Figure 3(a_1_). When the number of broadened rod s was constant, the widened width increased first and then decreased with the increase of the winding speed and the initial tension, and the interaction between *A* and *C* had no significant effect on the widened width, as shown in Figure 3(a_2_). When the initial tension was constant, with the increase of the winding speed and the number of straight rods, the broadened width increased first and then decreased, but the interaction between *B*_1_ and *C* had no significant effect on the broadened width, as shown in Figure 3(a_3_). In Figure 3a, the interaction of *A* and *B*_1_ had the greatest effect on the broadened width, followed by the interaction of *A* and *C*, and the interaction of *B*_1_ and *C* had the least effect on the broadened width.

The Box–Behnken test results are shown in Table 4. Equation (4) with broadened width (*w*_1_) as a response value and *A*, *B*_1_, and *C* as variables was obtained by regression fitting the experimental results:

(4) $$w_{1}=13.44+0.062\times A+2.46\times B_{1}+0.52\times C-0.38\times AB_{1}-0.25\times AC+0.05\times B_{1}C-0.43\times A^{2}-2.13\times {B_{1}}^{2}-0.76\times C^{2}$$

As shown in Table 5, the model $R_{Adj}^{2}$ was larger than the model $R_{Pred}^{2}$, i.e., 0.1539 < 0.2, and the signal-to-noise ratio was 16.731, which was >4, indicating that the model is reliable. The variance analysis of the regression model shows that the *p*-value of the model was <0.0001, indicating that the regression equation is very significant. The missing fitting item *p*-value of the model was 0.2809, which was >0.05, indicating that there are no other major factors affecting the response value, which further indicates that the model is relatively reliable. At the same time, the *p*-values of *B* and *B*^2^ were less than 0.0001, indicating that the factor had an extremely significant impact on the width of the developed fiber; the *p*-values of *C* and *C*^2^ were less than 0.05, indicating that the factor had a significant impact on the width of the developed fiber, as shown in Table 6.

#### 3.2.2. The Number of Convex Rods was 2

When the number of convex rods was 2 and the winding speed was constant, the initial tension had little effect on the broadened width. With the increase in the number of straight rods, the broadened width of the CF first increased and then decreased, as shown in Figure 3b. The interaction between *A* and *C* factors had a significant effect on the broadened width, as shown in Figure 3(b_2_). When the initial tension was unchanged, the broadened width first increased and then decreased with the increase of the number of straight rods, and the interaction between *B*_2_ and *C* had a significant impact on the broadened width, as shown in Figure 3(b_3_). In Figure 3b, the interaction of *A* and *C* had the greatest effect on the broadened width, followed by the interaction of *B*_2_ and *C*, and the interaction of *A* and *B*_2_ had the least effect on the broadened width.

The Box–Behnken test results are shown in Table 4. Equation (5) with broadened width (*w*_2_) as a response value and *A*, *B*_2_, and *C* as variables was obtained by regression fitting the experimental results:

(5) $$w_{2}=15.02-0.23\times A+3.46\times B_{2}+0.9\times C-0.038\times AB_{2}+1.42\times AC+1.12\times B_{2}C+0.23\times A^{2}-2.97\times {B_{2}}^{2}-0.18\times C^{2}$$

As shown in Table 7, the difference between the model $R_{Adj}^{2}$ and $R_{Pred}^{2}$ was less than 0.2, and the SNR of the model was 23.486, which was >4, indicating that the model is reliable. The variance analysis of the regression model shows that the *p*-value of the model was <0.0001, indicating that the regression equation is significant. The *p*-value of the model’s disfitting item was greater than 0.05, indicating that there are no other major factors affecting the response value. It further shows that the model is reliable. The effects of B_2_ and B_2_^2^ on the width of the developed fiber were extremely significant, and the effects of C, AC, and B_2_C on the width of the developed fiber were relatively significant, as shown in Table 8.

#### 3.2.3. The Number of Convex Rods was 3

When the number of convex rods was 3 and the number of straight rods was unchanged, the broadened width decreased first and then increased with the increase of the initial tension, as shown in Figure 3c. When the winding speed remained the same, the broadened width decreased first and then increased with the increase of the number of straight rods, and the interaction between *A* and *B* had a significant effect on the broadened width, as shown in Figure 3(c_2_). When the initial tension was constant, the broadened width first decreased and then increased with the increase of the number of straight rods, as shown in Figure 3(c_3_).

The Box–Behnken test results are shown in Table 4. Equation (6) with broadened width (*w*_3_) as a response value and *A*, *B*_3_, and *C* as variables was obtained by regression fitting the experimental results:

(6) $$w_{3}=10.5+0.19\times A-0.31\times B_{3}+0.38\times C-0.5\times AB_{3}-0.62\times AC+0.63\times B_{3}C+0.88\times A^{2}+1.13\times {B_{3}}^{2}-0.25\times C^{2}$$

Similarly, as shown in Table 9, the model is also reliable. The variance analysis of the regression model shows that the *p*-value of the model was *p* < 0.0001, indicating that the regression equation was significant, and the *p*-value of the model’s disfitting term was 0.5413, which was greater than 0.05, indicating that there are no other major factors affecting the response value. *AC*, *B*_3_*C*, *A*^2^, *B*_3_^2^ had a significant effect on the broadened width, while *A*, *B*_3_, *C*, *AB*_3_, and *C*^2^ had a significant effect on the broadened width, as shown in Table 10. 

In order to further validate the accuracy of the model, we could determine the optimal process conditions for 1, 2, and 3 convex rods through optimization analysis of the regression model and contour and response surface analysis of factor interactions. By comparing the actual values with the predicted values under these optimal conditions, it was evident that they were highly consistent, thus providing additional confirmation on the reliability of our model construction, as shown in Table 11.

### 3.3. Effects of the Broadening Process Parameters on Broadening Broadened Defects

#### 3.3.1. Effect on the Widened Gap

**Figure 4:**
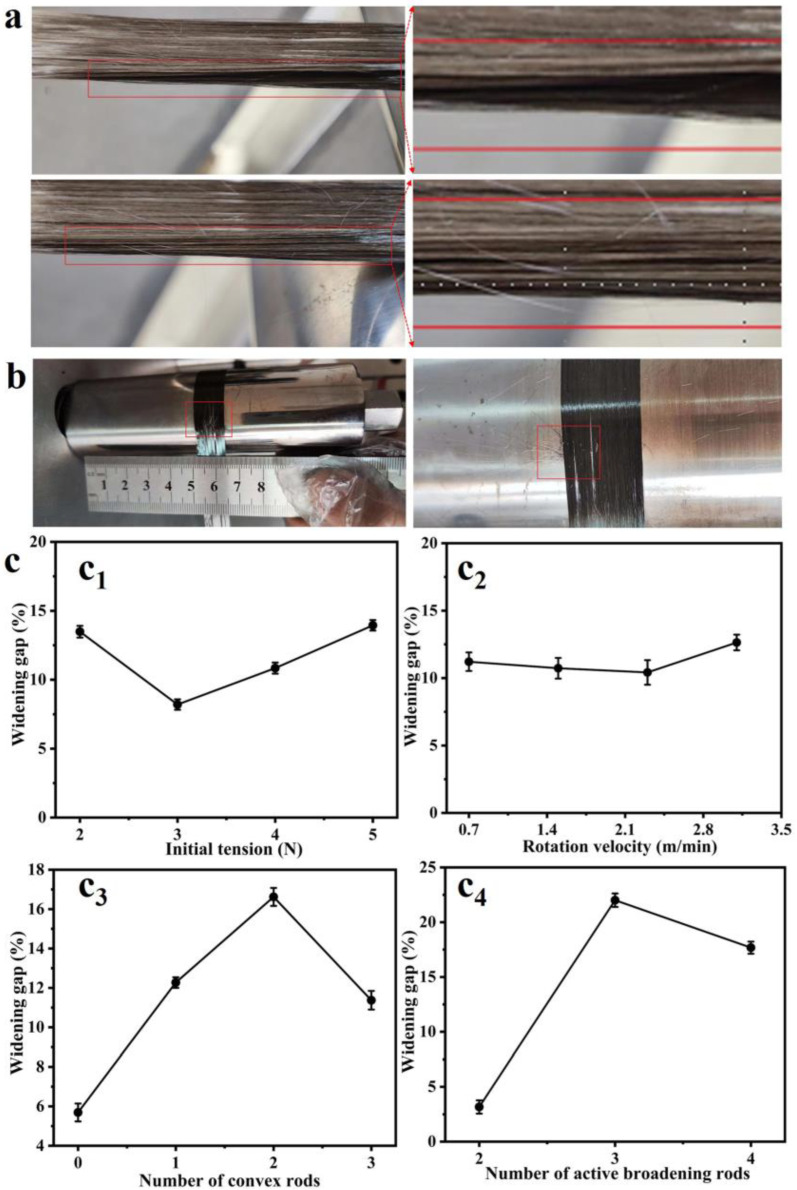
(**a**) Tow winding; (**b**) Widening the gap; (**c**) The probabilities of gaps in four different process parameters, namely (**c1**) initial tension, (**c2**) rotation velocity, (**c3**) number of convex rods and (**c4**) number of active broadening rods.

In the process of CF widening, a gap greater than 0.5 mm (Figure 4b) was generated due to tow winding (Figure 4a), which affected the uniformity and quality of CF tow widening [24,25]. In this study, the frequencies of the widened gap occurrence under different process parameters were counted, as shown in Figure 4c. As shown in Figure 4(c_1_), with the increase of the initial tension, the probability of a gap appearing in the CF tow during the broadening process first decreased and then increased. When the initial test tension was 3 N, the probability of a gap appearance was the lowest. As shown in Figure 4(c_2_), with the increase of the winding speed, the probability of a gap appearing in the broadening process fluctuated. When the winding speed was 2.3 m/min, the probability of at least one gap appearing in all experiments was the smallest. As shown in Figure 4(c_3_), the probability of having at least one gap increased first and then decreased with the increase in the number of straight rods. When one straight rod was used, the probability of having at least one gap was only 6.78%. As shown in Figure 4(c_4_), for the convex/straight rod combination adopted, the minimum probability of the gap occurrence when no convex rod was adopted was 5.21%. Therefore, the process parameters selected in the broadening process were an initial tension of 5 N, 0 straight rods, 3 convex rods, and a winding speed of 2.3 m/min, to reduce the widened gap in the broadening process as much as possible.

#### 3.3.2. Effect on Fiber Abrasion

**Figure 5:**
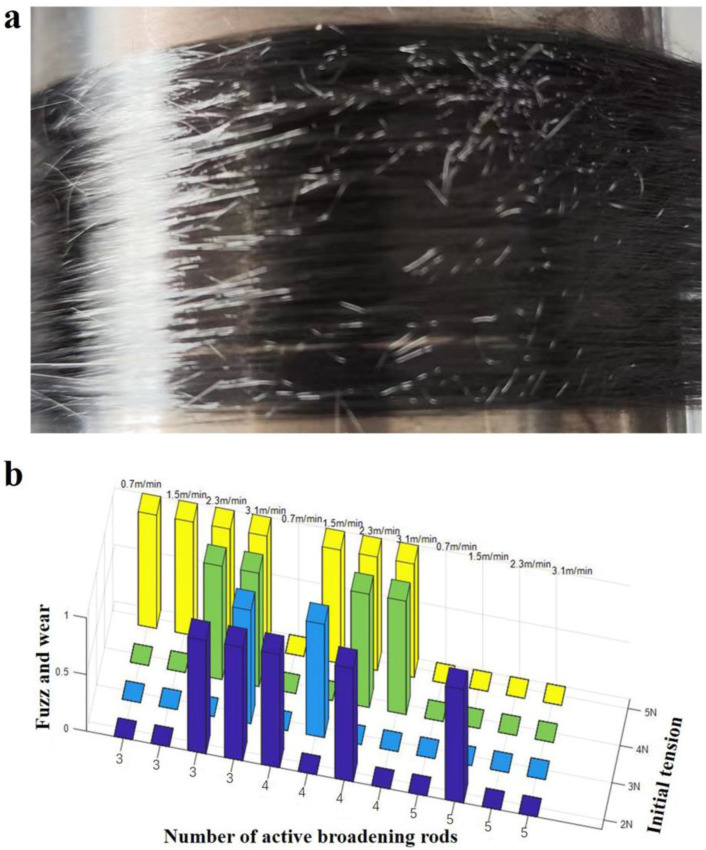
(**a**) Abrasion and fuzzing of the fiber bundle; (**b**) Fiber abrasion statistics under different parameters.

The friction between the CF tow and the surface of the broadening rod is dry friction. When the friction is too large, there are phenomena of fuzzing and single fiber fracture at the part of CF in contact with the broadening rod [26,27], as shown in Figure 5a. The fuzzing phenomenon caused by different process parameters that we can see with the naked eyes were called as “1”, and those without fuzzing were called as “0”. The statistical results are shown in Figure 5b. The friction between the fiber bundle and the rod was greater, and the abrasion was more serious when the initial test tension was larger. the more active broadening rods, and the faster the drawing speed.

#### 3.3.3. Effect on Fiber Breakage

**Figure 6:**
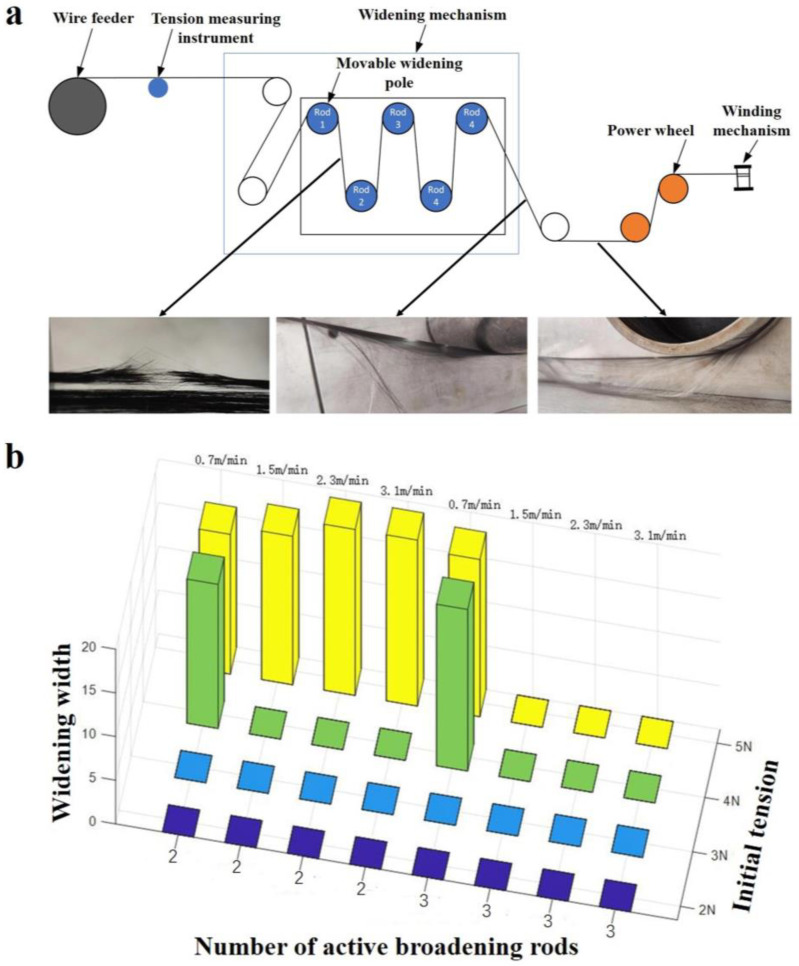
(**a**) Fracture location of the fiber bundle; (**b**) Fiber fracture statistics under different parameters.

During the widening process of the CF tow, fiber bundle breakage occurred at different positions, as shown in Figure 6a. If the number of active broadening rods was greater than 5 or the initial test tension was greater than 3 N, the fiber bundle fracture was caused. Under different process parameters, the fiber bundle fracture statistics are shown in Figure 6b. The fracture location was concentrated in the front and back of the tension wheel, and a small amount was found at the broadening rod, as shown in Figure 6a. According to the tribological theory, the relationship between the fiber tension after the broadening rod and the wrapping angle and the number of the broadening rod was shown in Formula (7):

(7) $$T={\left(T_{0}e_{\mu \varphi }\right)}^{n}$$

where

*T*_0_ is the initial tension (N);
*T* is the tension behind the rod (N);
*μ* is the coefficient of friction (N) between the CF tow and the broadening rod;
*φ* is the wrapping angle (rad) between the CF tow and the broadening rod;
*n* is the number of spreading rods.

According to Formula (7), with the increase of the number of active broadening rods and the initial tension, the tension borne by the CF tow during the broadening process also increases. When the limit of the tension borne by the CF tow was exceeded, the fiber bundle broke.

### 3.4. Analysis of Fiber Tensile Damage

**Figure 7:**
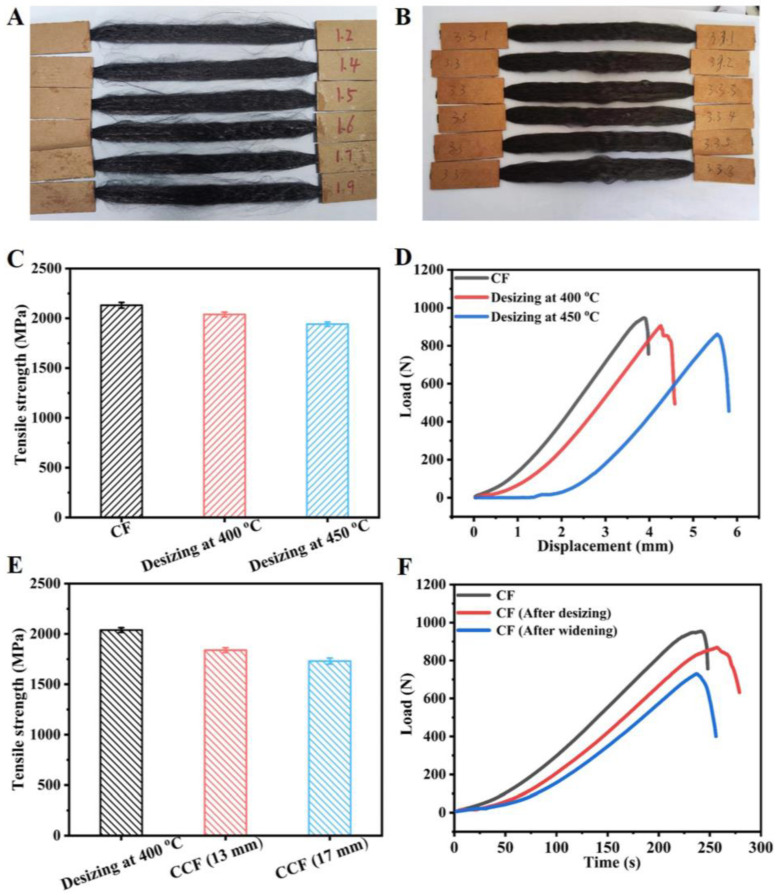
(**A**,**B**) Pictures of tensile test samples of CF strands and strands after desizing; (**C**,**E**) Tensile strengths of different specimens; (**D**) Mechanical properties of the CF tow under different temperature conditions; (**F**) Mechanical properties of the CF tow under different working conditions.

As shown in Figure 7C,E, the tensile strength of the fiber bundle was calculated after widening. The multi-filament tensile test of the CF showed that under the non-dipping treatment, the tensile strength of the CF was relatively low, and the fracture mode was single fiber bundle fracture, and no brittle fracture occurred in the whole fiber bundle. The mechanical properties of the fiber bundles obtained by different processing parameters are shown in Figure 7D. When the desizing temperature was 400 °C, the average tensile strength damage ratio of the desized tow to the original tow was 4.32%. When the desizing temperature was 450 °C, the average tensile strength damage ratio of the desized tow to the primary tow was 8.90%, mainly because under the action of an external load, the sizing agent on the surface of the CF was the first to be destroyed and the fibers began to be stressed only after the sizing agent was destroyed, while the fibers began to be stressed directly after desizing. Therefore, the tensile strength of the CF tow after desizing was less than that of the CF original tow. Since the damage to the CF tow was minimal at 400 °C, the desizing temperature was selected at 400 °C.

The fiber bundle fracture had the greatest effect on the mechanical properties of the CF during the broadening process, which led to the linear decline of the tensile strength of the CF. As shown in Figure 7E, as the widened width increased, it was more likely that a single fiber would break, resulting in a slight decrease in the widening of its overall fiber bundle. When the broadened width was 13 mm, the strength damage of the CF tow after broadening was 13.70% of that of the desized tow; when the broadening width was 17 mm, the strength damage of the CF tow after broadening was 18.78% of that of the desized tow, as shown in Figure 7F. Because the damage strength of 17 mm was large and it was easy to produce fuzzes and a widening gap, the broadened width of 13 mm was selected.

## 4. Conclusions

In this study, an experimental platform of automatic machinery widening was designed, and the effects of the process parameters on the width of the widening and the causes of the widening defects were analyzed. The results show that the widening effect can be improved by optimizing the process parameters, the widening can be increased, the widened gap can be reduced, the friction between the fiber and the broadening rod can be reduced, and the fracture of the fiber bundle can be reduced. When the initial tension of widening was increased, the speed of widening and drawing was too fast, and the number of active broadening rods was higher, widening defects were more likely to occur. At the same time, the best process parameters were obtained, such as the initial tension of 5 N, 0 straight rods, 3 convex rods, a drawing speed of 2.3 m/min, a broadened width of 13 mm with an effective broadening rate of 15.48%, and a widened CF tensile strength of 1839 MPa with a tensile strength damage ratio of 13.70%.

## Figures and Tables

**Table 1:** Physical and mechanical properties of the continuous carbon fiber for experiment.

Parameter	Value
Number of filaments	12 K
Tensile strength/MPa	2131
Tensile modulus/GPa	230
Elongation/%	2.1
Linear density/g/km	800

**Table 2:** CF tow widening factor design.

Initial Tension (N)	Number of Straight Rods	Number of Convex Rods	Rotation Velocity (m/min)
2	0	0	0.7
3	1	1	1.5
4	2	2	2.3
5	3	3	3.1

**Table 3:** Box–Behnken test factor values.

Factor	−1	0	1
A	2	4	5
B_1_	1	2	3
B_2_	0	1	2
B_3_	0	1	1
C	0.7	2.3	3.1

**Table 4:** Box–Behnken test results.

Number	A	B	C	Widened Width (mm)		
				1 Convex Rod	2 Convex Rods	3 Convex Rods
1	−1	1	0	14	16.15	12.5
2	0	0	0	13	14.5	10.3
3	1	−1	0	8.5	8.5	13.5
4	−1	0	−1	11	16	10
5	0	0	0	14	14.8	10.4
6	0	−1	−1	8	9	12
7	0	1	−1	12.5	13	10
8	0	0	0	13.1	15.3	10.7
9	−1	−1	0	8	8.5	12
10	0	0	0	13.6	15.5	10.6
11	0	−1	1	8.5	8.5	11.5
12	1	0	−1	12	12.3	11.5
13	0	1	1	13.2	13.2	12
14	1	1	0	13	13	12
15	0	0	0	13.5	13.5	10.5
16	1	0	1	13	13	11
17	−1	0	1	13	13	12

**Table 5:** Model parameters.

Parameter	Numerical Value	Parameter	Numerical Value
Standard deviation	0.47	$R^{2}$	0.9799
Signal-to-noise ratio	16.731	$R_{\mathrm{Adj}}^{2}$	0.9540
Mean value	11.88	$R_{\mathrm{Pred}}^{2}$	0.8001

**Table 6:** Box–Behnken regression model analysis of variance.

	Sum of Squares	Degree of Freedom	Mean Square	F	*p*-Value
Model	75.40	9	8.38	37.85	<0.0001
A	0.031	1	0.031	0.14	0.7182
B	48.51	1	48.51	219.15	<0.0001
C	2.21	1	2.21	9.96	0.0160
AB	0.56	1	0.56	2.54	0.1549
AC	0.25	1	0.25	1.13	0.3232
BC	0.010	1	0.010	0.045	0.8377
A^2^	0.79	1	0.79	3.56	0.1012
B^2^	19.15	1	19.15	86.50	<0.0001
C^2^	2.42	1	2.42	10.91	0.0131
Residual error	1.55	7	0.22		
Missing fit	0.90	3	0.30	1.84	0.2809
Pure error	0.65	4	0.16		
Total	76.95	16			

**Table 7:** Model parameters.

Parameter	Numerical Value	Parameter	Numerical Value
Standard deviation	0.51	$R^{2}$	0.9883
Signal-to-noise ratio	13.65	$R_{\mathrm{Adj}}^{2}$	0.9733
Mean value	23.486	$R_{\mathrm{Pred}}^{2}$	0.8715

**Table 8:** Box–Behnken regression model analysis of variance.

	Sum of Squares	Degree of Freedom	Mean Square	F	*p*-Value
Model	153.20	9	17.02	65.79	<0.0001
A	0.43	1	0.43	1.65	0.2394
B	95.57	1	95.57	369.36	<0.0001
C	6.48	1	6.48	25.05	0.0016
AB	5.625 × 10^−3^	1	5.625 × 10^−3^	0.022	0.8869
AC	8.12	1	8.12	31.39	0.0008
BC	5.06	1	5.06	19.57	0.0031
A^2^	0.23	1	0.23	1.89	0.3771
B^2^	37.05	1	37.05	143.19	<0.0001
C^2^	0.13	1	0.13	0.52	0.4942
Residual error	1.81	7	0.26		
Missing fit	1.18	3	0.39	2.51	0.1974
Pure error	0.63	4	0.16		
Total	155.01	16			

**Table 9:** Model parameters.

Parameter	Numerical Value	Parameter	Numerical Value
Standard deviation	0.15	$R^{2}$	0.9896
Signal-to-noise ratio	11.32	$R_{\mathrm{Adj}}^{2}$	0.9761
Mean value	0.9251	$R_{\mathrm{Pred}}^{2}$	30.486

**Table 10:** Box–Behnken regression model analysis of variance.

	Sum of Squares	Degree of Freedom	Mean Square	F	*p*-Value
Model	15.41	9	1.71	73.75	<0.0001
A	0.28	1	0.28	12.12	0.0103
B	0.78	1	0.78	33.65	0.0007
C	1.13	1	1.13	48.46	0.0002
AB	1.00	1	1.00	43.08	0.0003
AC	1.56	1	1.56	67.31	<0.0001
BC	1.56	1	1.56	67.31	<0.0001
A^2^	3.22	1	3.22	138.87	<0.0001
B^2^	5.33	1	5.33	229.55	<0.0001
C^2^	0.26	1	0.26	11.34	0.0120
Residual error	0.16	7	0.023		
Missing fit	0.062	3	0.021	0.83	0.5413
Pure error	0.100	4	0.025		
Total	15.57	16			

**Table 11:** Process parameters.

Convex Rod (Piece)	Initial Tension (N)	Straight Rod (Piece)	Winding Velocity (m/min)	Predicted Width (mm)	Practical Width (mm)
1	3	2	2.3	14.18	13.50
2	3	1	3.1	17.05	17.00
3	5	0	2.3	13.50	13.00

## Data Availability

The data presented in this study are available on request from the corresponding authors.
